# ER-Alpha-cDNA As Part of a Bicistronic Transcript Gives Rise to High Frequency, Long Term, Receptor Expressing Cell Clones

**DOI:** 10.1371/journal.pone.0031977

**Published:** 2012-02-20

**Authors:** Michal Shenfeld, Yafit Hachmo, Moran Frenkel, Naomi Dafni, Michael Boettcher, Joerg D. Hoheisel, Iris Dotan, Dan Canaani

**Affiliations:** 1 Department of Biochemistry and Molecular Biology, Faculty of Life Sciences, Tel Aviv University, Ramat Aviv, Israel; 2 Department of Functional Genome Analysis, Deutsches Krebsforschungszentrum, Heidelberg, Germany; Vanderbilt University Medical Center, United States of America

## Abstract

Within the large group of Estrogen Receptor alpha (ERα)-negative breast cancer patients, there is a subgroup carrying the phenotype ERα^−^, PR^−^, and Her2^−^, named accordingly “Triple-Negative” (TN). Using cell lines derived from this TN group, we wished to establish cell clones, in which ERα is ectopically expressed, forming part of a synthetic lethality screening system. Initially, we generated cell transfectants expressing a mono-cistronic ERα transcription unit, adjacent to a separate dominant selectable marker transcription unit. However, the yield of ERα expressing colonies was rather low (5–12.5%), and only about half of these displayed stable ectopic ERα expression over time. Generation and maintenance of such cell clones under minimal exposure to the ERα ligand, did not improve yield or expression stability. Indeed, other groups have also reported grave difficulties in obtaining ectopic expression of ERα in ERα-deficient breast carcinoma cells. We therefore switched to transfecting these cell lines with pERα-IRES, a plasmid vector encoding a bicistronic translation mRNA template: ERα Open Reading Frame (ORF) being upstream followed by a dominant-positive selectable marker (hygro^R^) ORF, directed for translation from an Internal Ribosome Entry Site (IRES). Through usage of this bicistronic vector linkage system, it was possible to generate a very high yield of ERα expressing cell clones (50–100%). The stability over time of these clones was also somewhat improved, though variations between individual cell clones were evident. Our successful experience with ERα in this system may serve as a paradigm for other genes where ectopic expression meets similar hardships.

## Introduction

Tumor expression of estrogen receptor alpha (ERα) plays an important role in the clinical care of breast cancer patients both as a prognostic factor and as a therapeutic target. Unfortunately, about two-thirds of breast cancer patients have an estrogen receptor alpha-negative disease. Within this large group of ERα^−^negative/endocrine therapy-resistant breast cancer patients, the Triple-Negative (TN) subgroup has bad prognosis, as it tends to develop metastases. So far, this group is being treated by surgery/irradiation and for the most part nonspecific chemotherapy [1].

Genes, whose activity, expression or dependence is considered to have increased in cancer, are prime candidates for therapeutic intervention. Cancer cells may depend upon such changes in gene expression, not only during tumor initiation, but also during malignancy progression (i.e. “oncogene addiction”). This is exemplified by the choice of the oncogene ERBB2/HER2 as drug target in ERBB2/HER2-positive breast cancer [2]. Alternatively, using the concept of synthetic lethality [3], efforts have been directed towards identification of chemicals/drugs or target genes whose activation or ablation, respectively, synergizes with mutations in either oncogenes or tumor suppressor genes [4], [5]. The availability of large-scale synthetic low-molecular-weight chemical libraries has allowed high-throughput-screening (HTS) for compounds that are synergistically lethal with defined human cancer aberrations in activated oncogenes or tumor suppressor genes; the so called “chemical synthetic lethality screens”. The generation of human/mouse genome-wide siRNAs and shRNA-expressing libraries has significantly advanced the complementing approach of “genetic synthetic lethality screen”. The latter is being performed either at the single gene level, in an array format, or primarily by retroviral/lentiviral-pools carrying shRNA expression cassettes that are used to infect target cells at low multiplicity of infection [6], [7]. In the case of the Triple-Negative derived BRCA1/BRCA2-deficient breast cancers, poly(ADP-ribose) polymerase (PARP), with or without DNA damaging agents, is synthetic lethal with BRCA1- or BRCA2-deficiency [8], [9]. Likewise, the frequent inactivation of the PTPN12 tyrosine phosphatase tumor suppressor gene in TN derived tumors renders them sensitive to inhibitors of multiple tyrosine kinases [10].

The first system toward which our groups have decided to apply the synthetic lethality screening approach entails ERα-negative breast carcinoma TN-derived cultured cells. In order to do so, one needs to test the specificity of the identified targets in an *in vitro* cell culture system. A compulsory control ingredient of the synthetic lethality screening in the ERα-deficient TN breast carcinoma cell lines is stable transfectants expressing the human ERα cDNA. In view of the heterogeneity observed in the TN breast cancer group, it is essential to generate such complemented systems in several different TN-derived cell lines.

In light of the difficulty in creating stable expression of ERα (see below), this manuscript offers an alternative methodology [11], [12] of doing so with greater success and fidelity. The generated ERα expressing clones can serve for the long term study of a variety of ERα associated topics.

## Methods

### A. Plasmids and constructs

pCDNA3-ERα, was constructed by the late Dr. Arnold Simons by first subcloning a 1820 bp SalI fragment encoding the complete coding sequence of wild type hERα from the GAL4 DB-hER plasmid [13] into the pBluescript II SK^−^ plasmid. Then the XhoI - HindIII fragment encoding the ERα sequence from BlueScript was cloned into the pCDNA3.3 a neo expression vector from Invitrogen. pCDNA3 by itself was named pCDNA3-empty, and used to construct the G418 resistant ERα non-complemented cell clones. The neo^R^ coding region is driven by the SV40 early genes promoter.

**Figure 1:**
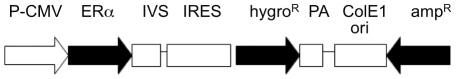
Map of pIRES-ERα bicistronic plasmid. The vector contains a single mammalian transcription unit initiating from the CMV immediate early promoter and terminating with an SV40 derived polyA addition fragment.

The pIRES-ERα plasmid (Fig. 1) is a derivative of the pIREShyg3 bicistronic vector (Clontech). The expression cassette of this vector contains the human cytomegalovirus (CMV) major immediate early gene promoter, followed by multiple cloning sites for cDNA/coding region insertion. A synthetic intron, is included downstream of the multiple cloning site. The encephalomyocarditis virus (EMCV) Internal Ribosome Entry Site (IRES) is followed by the bacterial hygromycin B resistance gene (Hygro^R^) and the SV40 polyadenylation signal. The coding sequence of the human ERα-cDNA was cloned downstream of the CMV promoter into the *EcoRV* site of PIREShyg3, as a blunted *EcoRV*-*XhoI* fragment.

The pCMV-Bam-ERα-Hygro was constructed first by deleting the *Bam*HI fragment encoding CD20 from pCMV-CD20 and religation of the vector. Next, an *Xba*I- *Hind*III fragment encoding TK-neo from the pCMV-Bam-neo was replaced with an *Nr*uI*-Sal*I fragment encoding for TK-hygro^R^ cassette from pCEP4 (Invitrogen). The coding sequence of human cDNA ERα was then cloned into pCMV Bam-Hygro by cloning an *EcoR*V-*Xho*I blunt-ended fragment encoding human ERα from pCDNA3-ERα (see above), into the *Bam*HI site of pCMV-Bam-Hygro.

The *firefly* luciferase reporter plasmid p2xERE-pS2-luc [14] was a kind gift from Prof. Y. Sharoni (Ben Gurion University) and the normalizing *renilla* pRNL-TK-luc (Promega) was a kind gift from Prof. L. Vardimon (Tel Aviv University).

### B. Cells growth

MDA-MB-231 [15] and GILM2 [16] were a kind gift from Prof. J. Price, MD Anderson. MDA-MB-435 and BT549 breast carcinoma cell lines were purchased from ATCC. MCF7 (ATCC) was a kind gift from R. Pinkas-Kramarski. Cell lines were routinely cultured at 37°C, 5% CO_2_, in DMEM supplemented with 5% fetal bovine serum (FBS), 4 mM L-glutamine, and penicillin/streptomycin; these five medium ingredients were purchased from Biological Industries (Israel). ERα transfected cell clones were maintained in phenol red-free DMEM medium (Biological Industries, Israel) supplemented with 5% dextran coated Charcoal Stripped fetal calf Serum (CSS, manufactured by Hyclone, US) to prevent ERα activation (see below).

### C. Cell transfection and clonal selection

MDA-MB-231, MDA-MB-435, and GILM2 transient and stable transfections were carried out using jetPEI reagent (PolyPlus Transfection, France) according to the manufacturer's instructions. In order to produce stable clones, a 1∶5–1∶20 dilutions of 5×10^6^ transfected cells was performed into 100 mm Petri dishes 48 hours post transfection. Selection was commenced the day after. Selective media consisted of DMEM without phenol-red, supplemented with 5% dextran charcoal fetal bovine serum (FBS), 4 mM L-glutamine, antibiotics (10 units/ml of penicillin and 50 µg/ml streptomycin) and the selective drug. Selection of stable clones was performed at 0.4 mg/ml G418 (Calbiochem) for pCDNA3-neo based clones, or at 0.2 mg/ml Hygromycin B (A.G. Scientific) for pIREShyg3 and pCMV-Bam-ERα-Hygro^R^ based clones. Selective media was refreshed every 3 days thereafter. When colonies were big enough and interspaced, they were transferred to 48- well cell culture plates. For long term maintenance, 0.2 mg/ml G418, or 0.1 mg/ml Hygromycin B were used.

### D. Western blot analysis

MCF7, BT549, MDA-MB-231, MDA-MB-435, GIML2 and their clonal derivatives were washed twice with cold Hanks buffer (Biological Industries, Israel), scraped with a rubber policeman and collected to a new tube. The cells were then centrifuged at 2000 rpm, 4°C for 5 minutes and pellets were lysed in ice-cold modified RIPA buffer (1% NP-40, 50 mM Tris pH 8, 0.15 M NaCl, 5 mM EDTA, 0.5% DOC and 1 mM PMSF, without SDS). Lysates were incubated on ice for 10 minutes, then cleared by centrifugation and stored in −70°C until use. For the Western blot analysis, the protein of each cell lysate was quantified by using the Bradford assay. 50 µg of each lysate was diluted 1∶2 with a 4× SDS-PAGE sample buffer to a final concentration of 2× SDS-PAGE sample buffer (0.12 M Tris-Cl pH 6.8, 4% SDS, 20% glycerol, 0.2 M DTT, 0.008% bromophenol blue). These lysates were denaturated and separated on 10% polyacrylamide gel at 100v for 90 minutes at room temperature. Proteins were transferred to nitrocellulose membranes (BioScience, Germany) by electroblotting for 120 minutes at 12–20v or 120–150 mA. Membranes were blocked with blocking solution- 1% nonfat dry milk in PBS-T (137 mM NaCl, 2.7 mM KCl, 4.3 mM Na_2_HPO_4_ containing 0.1% tween) 20 for 1 hour at room temperature. The membranes were then probed with hERα mouse monoclonal primary antibody (NCL-ER-6F11; Novocastra Labs Ltd, England) at 1∶1000 dilution in blocking solution overnight at 4°C, followed by 3×5 min washes in PBS-T 0.1%. Next, the membranes were incubated for 1 hour at room temperature with Goat anti mouse IgG HRP conjugated secondary antibody (Sigma, Israel), at 1∶5000 dilution in blocking solution. Next, 3×5 min washes were preformed and the membranes were incubated with a home-made chemiluminescence solution (ECL solution–100 mM Tris, pH 8.5, 1.25 mM luminol, 0.2 mM p-cumaric acid, 0.01% H_2_O_2_) for 1 minute. Blots were then exposed to film (Kodak) and developed. Signal quantization was performed by densitomentric analysis using a GE ImageQuant 350 scanner. After antibody stripping, α-tubulin was probed with a mouse monoclonal antibody (Sigma, Israel) at a dilution of 1∶5000 and used as a cellular normalizing marker.

### E. Dual-luciferase reporter assay

In order to assay ERα activity, cells were seeded in 24-well cell culture plates at 50–70% density, in DMEM supplemented with 5% FCS. The next day, the cells were transiently co-transfected with 0.5 µg p2xERE-PS2-luc (primary reporter vector containing the firefly luciferase gene under the ERα Response Element; i.e. ERE) and 0.3 µg pRNL-TK-luc (secondary reporter vector containing the *Renilla* luciferase gene under the constitutive HSV TK promoter), using the jetPEI transfection reagent. Forty-eight hours post-transfection, cells were washed twice with Hanks' (Biological industries), a balanced salts solution without phenol red, and cell lysates were prepared as described in the manufacturer's protocol for dual-luciferase reporter assay (Promega, USA). Briefly, cells were lysed with 45 µL/well of Passive Lysis Buffer for 10 minutes at room temperature. The firefly luciferase assay was initiated by adding 5–15 µL aliquot of cell lysate to 50 µL of Luciferase Assay Reagent II (LAR II). After recording the luminescence, 50 µL of Stop & Glo reagent was added to the same tube in order to quench the firefly luciferase reaction and simultaneously activate the *Renilla* luciferase reaction. Firefly and *Renilla* luciferase activities were measured using a LKB Wallac 1250 Luminometer. The firefly luciferase luminescence measured was proportional to the amount of active ERα protein present in the cells. The *Renilla* luciferase luminescence was proportional to the efficiency of the transfection. This internal control provides a convenient and reliable assay of efficiency. Normalized luciferase luminescence was calculated as followed: [(firefly luciferase activity/*Renilla* luciferase activity)×100]. These results, determined from lysates ERα complemented clones (as well as the positive and negative control), were then normalized again to MCF-7 positive control by dividing them to the same ratio obtained from the positive control: [(firefly/*Renilla* luminescence×100)/(MCF-7 firefly/*Renilla* luminescence×100)×100]. All experiments *were performed several times in duplicates.*

### F. RT-PCR

For expression confirmation originating from the pIRES-ERα construct, RT-PCR was conducted. Two µg of total RNA extracted using EZ-RNA isolation kit (Biological Industries, Israel) were transcribed into first strand cDNA by hexamer priming, followed by PCR reactions as specified in the Long range RT-PCR kit (Qiagen). The PCR conditions included preincubation for 3 minutes at 93°C and 40 cycles comprised of 30 seconds at 93°C, 30 seconds at 54°C, 4.5 minutes at 68°C, and finishing up 10 minutes at 68°C.

PCR primers (Hylabs, Israel) were as follows:

ERα:

Sense 5′-ATGACCATGACCCTCCACAC-3′,

antisense 5′-AGACTGTGGCAGGGAAACC-3′

Hygromycin B:

sense 5′- CTGTCGAGAAGTTTCTGATCG-3′

antisense 5′- AGTACTTCTACACAGCCATCG-3′

### G. Estimation of the cell growth doubling time

Each clone was seeded at a density of 2.5–3×10^4^ cells in 24-well tissue culture plates, and was incubated at 37°C in 5% CO_2_. The cells were counted every day for 5–6 days, using a cell counting chamber (Hemocytometer). The doubling time of each clone was calculated as following: [2×24 hours/(Ave (no. of cells in day (X+1)/no. of cells in day X)].

## Results

### A. Generation & characterization of ERα-expressing MDA-MB-231 stable transfectants with the pCDNA3-Erα expression vector

#### A1. Transfection and selection of ERα expressing clones in MDA-MB-231 cells

In order to establish a supporting control system for synthetic lethality screening of ERα-negative breast cancer cells (of the TN subgroup), two human epithelial breast carcinoma cell lines, BT549 and MDA-MB-231, were initially utilized as recipients for the ERα-expressing constructs. The MDA-MB-231 cell line was particularly suitable for such preliminary studies since it is highly aggressive both *in vitro* as cell culture and *in vivo* upon grafting [15].

These two cell lines were initially transfected with pCDNA3-ERα. Simultaneously, these cell lines were also stably transfected with the pCDNA3 vector by itself, to serve as a negative control (pCDNA3-empty). Subsequent selection with G418 resulted in the establishment of the two groups of stable cell clones, ERα-complemented and ERα-empty (non-complemented). Initial studies performed in the presence of DMEM medium supplemented with 5% FCS showed that similar to other groups [17], [18], the ERα complemented clones were much harder to establish than the empty vector control group. This observation was also reminiscent of studies demonstrating that ERα expression following long-term estrogen deprivation in ERα-positive breast cancer cells is thereafter manifested by an initial phase of estrogen hypersensitivity. This phase is characterized by apoptosis and rapid tumor regression at concentrations of estrogen (E_2_, Sigma Israel) below 10^−13^ M [19].

For these reasons, we decided to attempt generating the ERα-complemented MDA-MB-231 clones (which in some way are analogous to E_2_ deprived ERα positive cells) in DMEM without phenol red, supplemented with 5% CSS. This way, exposure to residual ERα-receptor-activating agents was minimized, making the clones less sensitive to the ectopic expression of ERα.

In order to examine whether estrogen deprivation affects the stability and long term expression of functional ERα, newly emerging clones were grown simultaneously in e the regular phenol red-free DMEM supplemented with 5% CSS, as well as in DMEM supplemented with 5% FCS.

**Figure 2:**
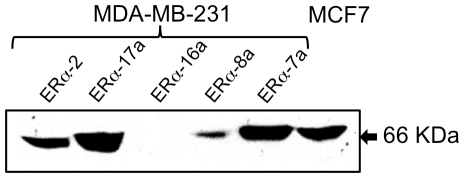
pCDNA3-ERα transfectants of MDA-MB-231: Western immunoblot analysis of ERα expressing clones. The MCF-7 cell line was used as an ERα-positive control.

Forty established MDA-MB-231 cell clones selected for G418 resistance were then tested for ERα expression by Western blot analysis. Fig. 2 shows that only few clones (ERα-2, ERα-7a, ERα-8a and ERα-17a) of the established cell clones, express the protein. MCF7, a *bona fide* ERα-positive cell line, was used as positive control. ERα-16a, a cell clone which was established after transfection with pCDNA3-ERα integrating plasmid, turned out not to express the protein. It also served us as a negative control, when required. The four clones show various levels of ERα expression, as compared to the positive control (Fig. 2).

#### A2. Selecting for ERα-active clones

The next phase was to analyze whether the selected clones synthesizing the ERα, protein express a functional receptor. The quantification of ERα activity was performed by the dual luciferase reporter assay (see Methods).

MDA-MB-231 established clones were initially tested while grown in DMEM supplemented with 5% FCS, which naturally contains estrogen (E_2_).

**Figure 3:**
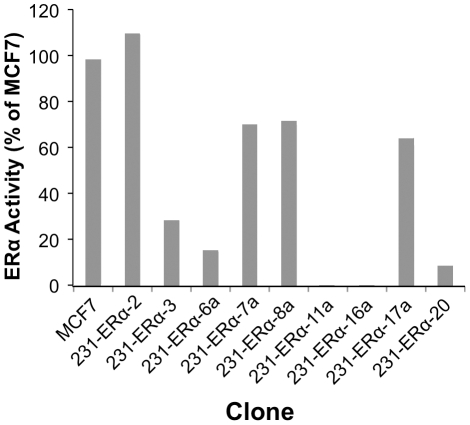
pCDNA3-ERα transfectants of MDA-MB-231: Dual-Luciferase reporter assay for ERα activity. G-418 resistant cell clones were seeded in 24-well tissue culture plates at 50–70% density in DMEM supplemented with 5% FCS. Twenty four hours later, cells were transiently transfected with the pERE-PS2-luc plasmid together with the pRNL-TK plasmid. Forty eight hours after transfection, cells were lysed whereby *firefly* and *Renilla* luciferase activities were measured and normalized to the positive control, MCF7.

Fig. 3 summarizes the results obtained from different MDA-MB-231 established clones. Four clones; 231-ERα-2, 231-ERα-7a, 231-ERα-8a and 231-ERα-17a, exhibited 66% to 111% of the expression level displayed by the positive control MCF7. Five clones; 231-ERα-3, 231-ERα-6a, 231-ERα-11a, 231-ERα-16a and 231-ERα-20, expressed between 0% and 30% receptor activity, as compared to MCF7. Not only were these levels very low, but further ERα reporter assays showed that these five clones continued loosing activity over time. Additional tests performed on the former four ERα expressing clones, showed maintenance of appreciable levels of activity despite fluctuations over time (see below).

#### A3. Responsiveness to ligand

**Figure 4:**
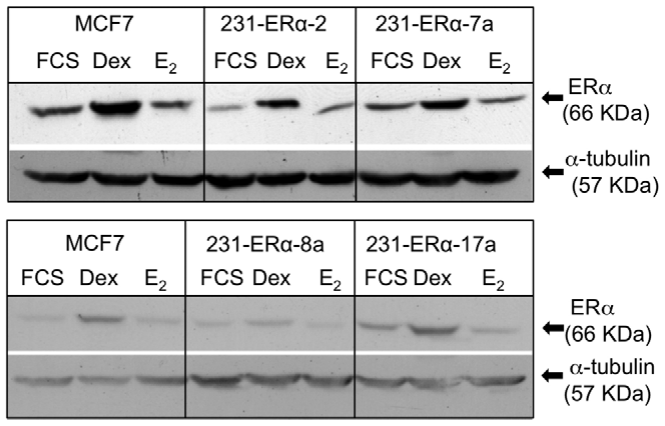
pCDNA3-ERα transfectants of MDA-MB-231: Responsiveness to ligand. MDA-MB-231 derived clones were seeded in 60 mm dishes and grown for 24 hrs under three conditions: DMEM supplemented with 5% FCS, phenol red-free DMEM supplemented with 5% CSS, and phenol red-free DMEM supplemented with 5% CSS and 2×10^−8^ M E_2_. The top panel shows the 66 KDa ERα protein detected with the anti-hERα antibody. The bottom panel shows the 57 KDa α-tubulin protein within the same blot after stripping the anti-hERα antibody and re-probing with the anti-α-tubulin antibody.

The next step was to determine whether the ectopically expressed ERα was under hormonal regulation. Several studies have shown that adding estrogen to serum starved ERα -positive cells, or to ERα ectopically expressed cells, can down-regulate expression of the receptor. The decrease requires a functional receptor and occurs at both the protein and mRNA levels [19], [20]. This phenomenon has led us to systematically examine our ERα-complemented clones for responsiveness to regulation by estrogen. In order to do so, MCF-7 cells and four ERα complemented clones; 231-ERα-2, 231-ERα-7a, 231-ERα-8a and 231-ERα-17a, were seeded in 60 mm dishes under three different growth conditions: DMEM supplemented with 5% FCS, phenol red-free DMEM supplemented with 5% dextran coated charcoal filtered FCS (Dex), and phenol red-free DMEM supplemented with 5% dextran coated charcoal filtered FCS and 2×10^−8^ M E_2_. After 24 hours, expression of ERα in these clones, under the three conditions was determined by Western immunoblot analysis. Fig. 4. reveals that all dishes treated with E_2_ expressed a lower level of ERα compared with the parallel estrogen starved cells (Dex). As also expected, dishes treated with 5% FCS (FCS) expressed a lower level of estrogen receptor compared to the Dex cells, in accordance with estrogen saturating levels found in FCS.

**Figure 5:**
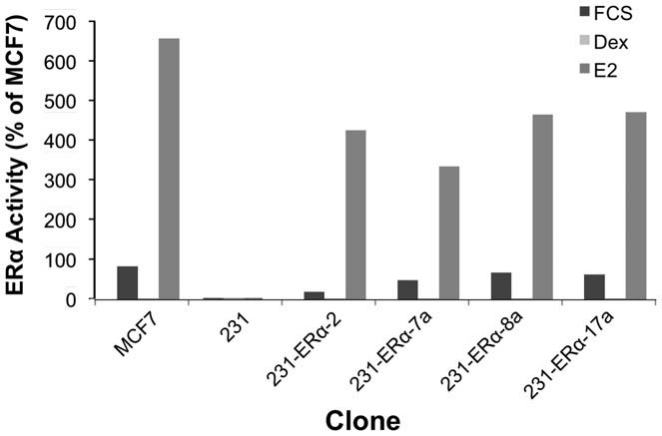
pCDNA3-ERα transfectants of MDA-MB-231: Testing Response to ERα ligand via the Dual-Luciferase reporter assay. Clones were plated in 24-well tissue culture plates at 50–70% density under the three growth conditions mentioned in the legend to Fig. 4. Twenty four hours later cells were transiently co-transfected with a p2xERE-pS2-luc plasmid together with a pRNL-TK plasmid. Forty eight hours after transfection cells were lysed whereby *firefly* and *Renilla* luciferase activities were measured and compared to the positive control, MCF-7. MDA-MB-231 parental cell-line was used as a negative control. The presented values were normalized to that of MCF-7 cells seeded in DMEM supplemented with 5% FCS.

Cell clones responding to the ligand regulation were also assayed for receptor activity under the different treatments. In order to do so, MDA-MB-231 established clones were seeded in 24-well tissue culture plates at the three different growth medium conditions, as mentioned above. Luciferase reporter plasmids were then transfected. After 24 hours, cell extracts were prepared and assayed. Fig. 5 summarizes the results obtained from these clones, comparing them to the positive control MCF7, grown in FCS.

When cell clones were seeded in DMEM supplemented with 5% FCS, they exhibited expression levels of 35% to 85% as compared to the expression of the positive control MCF7, which was assigned 100% relative activity. When cells were seeded in phenol red-free DMEM supplemented with 5% CSS, they behaved similarly to MCF7 and manifested an insignificant level of active ERα, in line with absence of the ligand (E_2_). Naturally, the receptor was not activated, leading to its inability to bind to the ERE in p2xERE-pS2-luc reporter. However, when cells were seeded in phenol red-free DMEM supplemented with 5% CSS treated with 2×10^−8^ M added E_2_, a significant increase in the activity level was exhibited.

Obviously, MDA-MB-231 parental cell-line did not display any significant expression level under all three conditions, since there is no ERα to be activated in the first place.

#### A4. Stability of the cell clones

**Figure 6:**
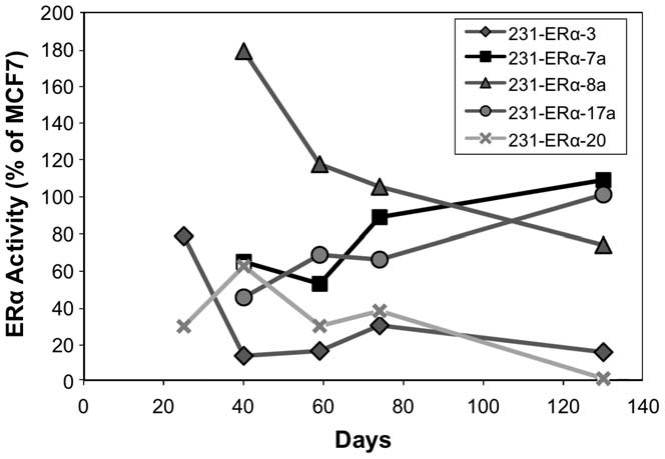
pCDNA3-ERα transfectants of MDA-MB-231: Assaying stability of ERα trans-activity via the dual luciferase assay. For each time point, measured in days from the received time of individual clones covering a 60 mm plate, cells were plated in 24-well tissue culture plates at 50–70% density, and grown in DMEM supplemented with 5% FCS. Twenty four hours later, cells were transiently co-transfected with a p2xERE-pS2-luc plasmid together with a pRNL-TK plasmid. Forty eight hours after transfection cells were lysed whereby *firefly* and *Renilla* luciferase activities were measured and normalized to the positive control, MCF-7.

In order to determine the clones' stability over time, ERα activity was assayed periodically via the dual luciferase reporter assay. The dual luciferase activity values obtained were normalized to the activity obtained in MCF7, transfected at the same time point, alongside the clones. According to Fig. 6, three clones; 231-ERα-7a, 231-ERα-8a, and 231-ERα-17a, maintained an intermediate activity level (40–90% of MCF7 level) over at least 130 days. Two clones; (231-ERα-3 and 231-ERα-20) displayed relatively low activity, which eventually (day 130) decayed.

As mentioned above, clones established from the parental MDA-MB-231 cell line were maintained in culture with DMEM supplemented with 5% FCS, but also in phenol red-free DMEM supplemented with 5% CSS. We did not observe any difference in the cell clones' stability of receptor activity under the two growth conditions (data not shown).

Because drug administration efficiency is affected by the cultured cells' proliferation rate, we wished to compare the growth rate of MDA-MB-231 cell clones expressing ERα. As it turned out, the doubling time of two ERα complemented vs. four ERα non-complemented (empty) clones was similar (around 21 hours), with almost identical growth curves (data not shown).

### B. Generation and characterization of MDA-MB-231 stable transfectants with ERα expressed from a bicistronic transcription unit

#### B1. Transfection & selection

As outlined above, the yield of MDA-MB-231 stable cell clones expressing ERα, following stable transfection and G418 selection with the monocistronic pCDNA3-ERα vector was rather low (12.5%, 5/40). Also, ERα expression was somewhat unstable over time. Large-scale experiments with the same vector in the triple negative breast carcinoma cell line BT549 resulted similarly in only five out of forty cell clones expressing a ligand responsive hormone receptor (Moran Gilat, M.Sc. Thesis Tel Aviv University 2006). Moreover, our other unpublished studies which we performed with MDA-MB-231 cells transduced with another monocistronic vector, pCMV-Bam-ERα-Hygro (whose stable selection for Hygromycin B resistance is driven by the strong HSV TK promoter), resulted in 48 cell clones, only four of which express the ERα receptor (Lilach Wallerstein-Shomrony M.Sc. Thesis Tel Aviv University 2006). These inefficient attempts to recover ERα expressing cell clones had initiated the trial to establish an improved ectopic expression system using a bicistronic mRNA template for ERα translation (Fig. 1). The vector consists of a single transcription unit having the ERα ORF as the upstream cistron, and a dominant-positive selectable marker (Hygro^R^), forming the downstream cistron, translated from an Internal Ribosome Entry Site (IRES).

This configuration has the advantage that selection for the IRES-directed selectable marker gene expression may protect the transcription unit as a whole, including the upstream ERα ORF. Thus, this linkage may lead to a high yield of ERα-expressing clones. Accordingly, MDA-MB-231 parental cells were stably transfected with the ERα-IRES construct (Fig. 1). Transfection and selection were performed under minimal estrogen growth conditions, where phenol red-free DMEM medium supplemented with 5% CSS was used.

**Figure 7:**
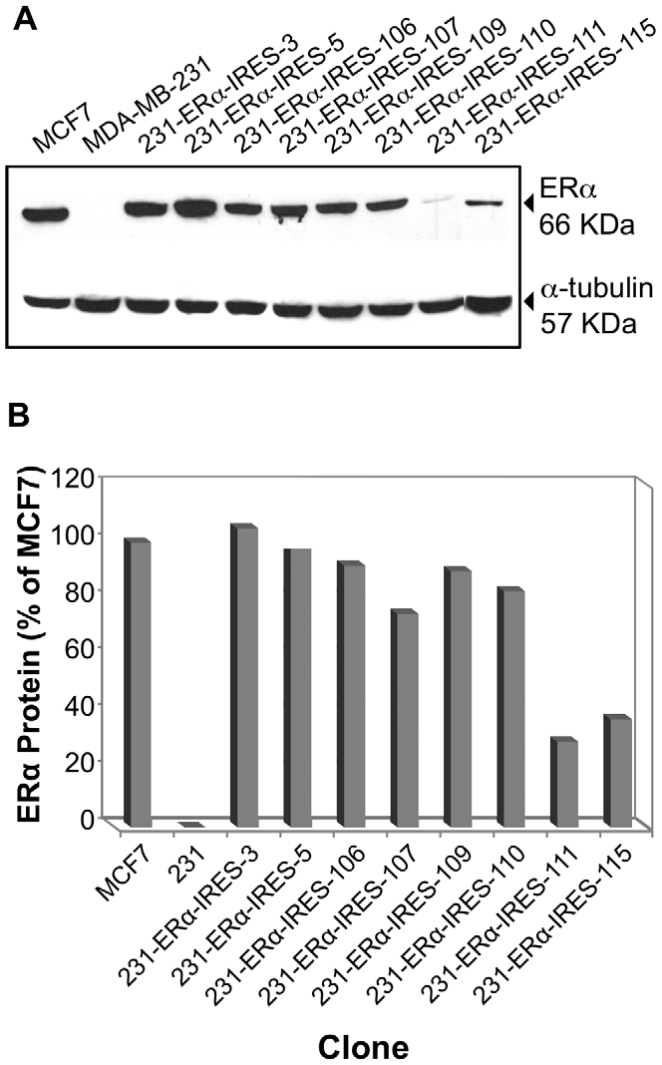
pERα-IRES MDA-MB-231 transfectants: ERα expression via Western immunoblot analysis. **A.** MDA-MB-231 clones selected for Hygromycin B resistance were lysed and ERα expression was tested by Western immunoblot analysis. ERα positive MCF-7 cell line was used as a positive control. MDA-MB-231 parental cell-line represented the negative control. **B.** Representation of ERα steady state expression values. The values of ERα expression were normalized to tubulin expression, with MCF7 value being one unit.

Screening of Hygromycin B resistant clones for ERα expression was initially performed by Western immunoblot analysis. Fig. 7A & 7B show the results obtained with the different cell clones.

**Figure 8:**
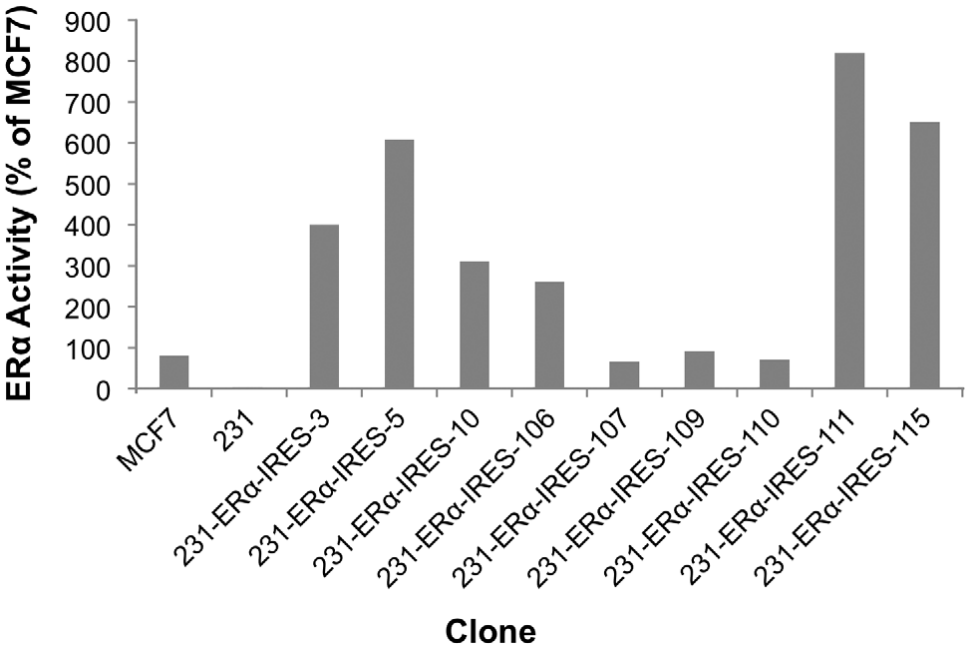
pERα-IRES MDA-MB-231 transfectants: Dual-Luciferase reporter assay for ERα activity. Technical details as in the legend to Fig. 6.

Surprisingly, all hygromycin B resistant clones (8/8) showed some level of ERα expression. Upon testing these clones for ERα activity by the dual luciferase reporter assay, it became evident that the high frequency of ERα protein expression in the selected clones is accompanied by ERα activity (Fig. 8). Yet, as also observed by others, the relationship between Immunoblot quantification and activity is not always linear, for various potential reasons such as misfolding of the protein or proteolytic cleavage of terminal amino acids leading to loss of activity, etc.

Nevertheless, all nine IRES-ERα descendant clones of the MDA-MB-231 parental cell line showed high ERα-mediated activation of the reporter gene, amounting from 85% to 841% (!) of the level displayed by MCF7.

**Figure 9:**
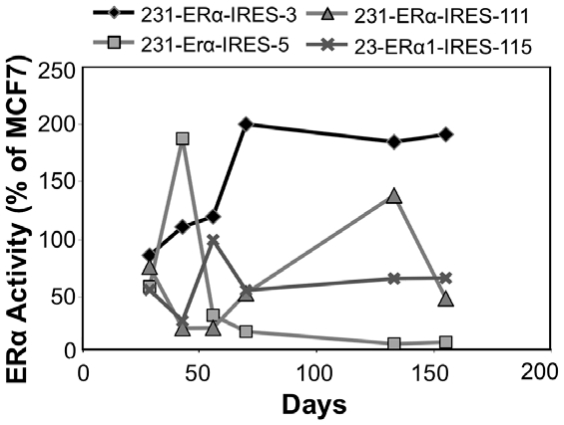
pERα-IRES MDA-MB-231 transfectants: ERα activity over time. Technical details as in the legend to Fig. 6.

In order to evaluate stability of ERα expression over time, dual-luciferase assays were performed intermittently over a relatively long time period (Fig. 9). Cell clones were kept under Hygromycin B selection, in phenol red-free DMEM medium supplemented with 5% CSS, in order to minimize potential expression suppression by the ligand. At each time point of assay, dual luciferase activity was normalized to the activity obtained in MCF7, transfected at the same time point, alongside the clones.

Examination of the stability of the ectopically expressed ERα-IRES clones in MDA-MB-231 over time (Fig. 9), revealed that three out of four clones still retain appreciable activity (as compared to that of the reference, MCF7 cells) 155 days after being initially monitored, amounting to over six months post transfection with the pERα-IRES vector.

#### B2. Characterization of the ERα-IRES-Hyg^R^ containing transcript

After obtaining a high yield of ERα expressing cell clones among the hygromycin B resistant transfectants in MDA-MB-231 (9/9), we set to characterize the ERα-containing hybrid transcript. A 3.2 kb fused transcript, encoding both cistrons: the ERα and the hygromycin B resistance selectable marker gene, was anticipated. Noteworthy, the plasmid sequence contains an intron situated downstream of the ERα ORF in such a way that splicing of the intron would result in RT-PCR product shorter by 295 bp (3.2 kb), compared to the plasmid template PCR product (3.5 Kb).

**Figure 10:**
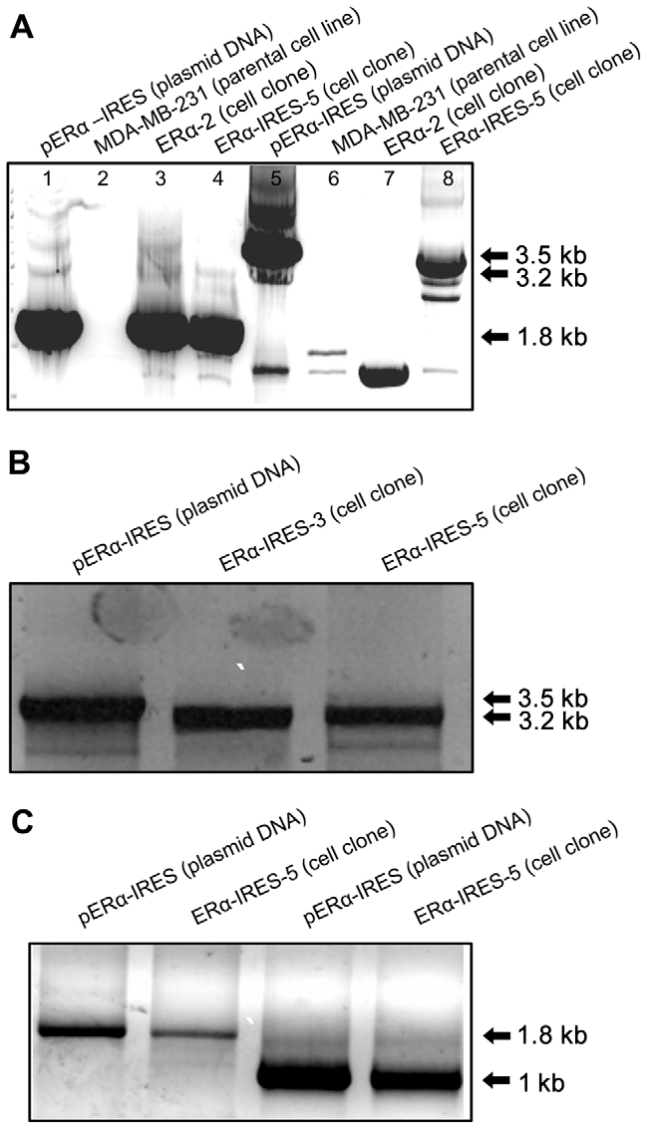
Characterization of ectopically expressed RNAs by long range RT-PCR. MDA-MB-231 parental cell line (231-parental), its pcDNA3-ERα stable transfectant (ERα-2), and its ERα-IRES stable transfectants (ERα-IRES-5 and ERα-IRES-3) were analyzed for expression of ERα–harboring transcript (1.8 kb), Hygromycin B resistance gene-containing transcript (1.0 kb), and ERα-IRES-Hygro^R^ fused transcript (3.2 kb), by RT followed by long range PCR amplification. pERα-IRES DNA served as a PCR positive control for the ERα cDNA primers (1.8 kb), the Hygromycin B resistance gene ORF primers (1.0 kb), and the 5′ sense ERα primer plus 3′ antisense Hygro^R^ fused ORFs primers (3.5 kb). **A** First four lanes from left contain the ERα cDNA primers; lanes 5–8 the 5′ sense ERα primer together with the 3′ antisense Hygro^R^ gene primer. **B.** The 5′ sense ERα primer together with the 3′ antisense Hygro^R^ gene primer. **C.** Lanes 1 and 2 from left, the ERα primers. Lanes 3 and 4 the Hygro^R^ gene primers. Primer sequences are detailed in the “Methods” section.

Lane 8 of Fig. 10A and the second and third lanes from left of Fig. 10B show that in two representative clones: ERα-IRES-5 and ERα-IRES-3, the expected 3.2 kb RT-PCR product of the spliced fused transcript is amplified. In contrast, ERα-2 a cell clone, described above and created by the monocistronic construct pCDNA3-ERα and devoid of the Hygromycin selectable marker, expressed the 1.8 kb ERα RNA product (lane 3 of Fig. 10A). The ERα-2 cell clone was used as a positive control for the ERα RT-PCR reaction, since based on prior Northern blot analysis, we observed that it contained an intact mRNA of the active ERα protein (data not shown). Thus, we have demonstrated in our MDA-MB-231 cell clones the presence of a full-length fused ERα-IRES-Hygro^R^ transcript, which is also correctly spliced; see in Fig. 10B for example the PCR product size from pERα-IRES (3.5 kb) vs. the RT-PCR product from RNA belonging to each of the two ERα-IRES cell clones (3.2 kb).

#### B3. Generating stable transfectants with ERα expressed from a bicistronic transcription unit in MDA-MB-435 & GILM2 cells

The high yields (9/9) of ERα expressing cell clones in the TN-derived ERα-deficient breast carcinoma cell line MDA-MB-231 stands in contrast to previous studies carried out in our laboratory (and described in part above), as well as those of others (mentioned before), in which the efficiency of generating stable ectopic ERα expressing breast carcinoma cells is a cumbersome and inefficient procedure, resulting in a mere ∼5% valuable clones.

In view of the vast genetic heterogeneity within breast carcinomas, and the triple-negative breast cancer patients' group in particular [21]–[24], there is demand for generating additional ectopic ERα producers.

Accordingly, we decided to attempt the bicistronic vector approach in two more triple-negative breast cancer cell lines: MDA-MB-435 [15], [25], and GILM2 [16]. Both cell lines were transfected with the pERα-IRES construct as before and selection with hygromycin B was carried out under minimal estrogen exposure conditions, as previously outlined.

**Figure 11:**
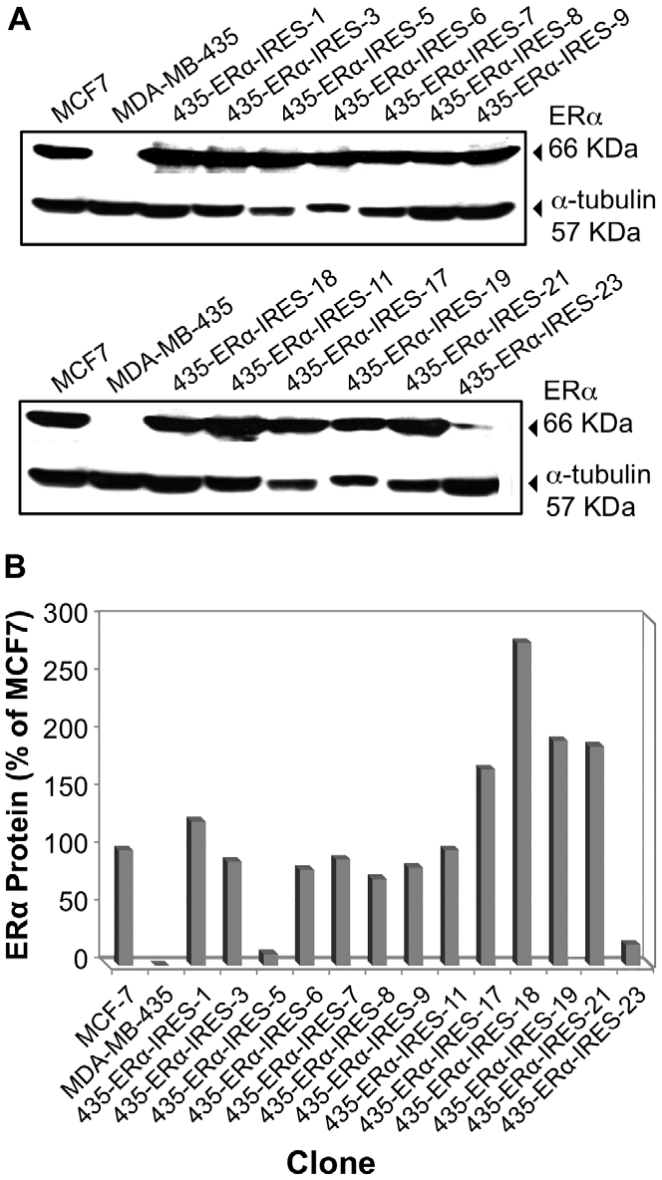
pERα-IRES MDA-MB-435 transfectants: Western immunoblot analysis of ERα protein. **A.** MDA-MB-435 cell clones selected for Hygromycin B resistance were lysed and ERα expression was tested by Western immunoblot analysis. ERα positive MCF-7 cell line was used as a positive control. MDA-MB-435 parental cell-line represented the negative control. **B.** Representation of ERα steady state expression values. The values of ERα expression were normalized to α tubulin expression in the cells.

**Figure 12:**
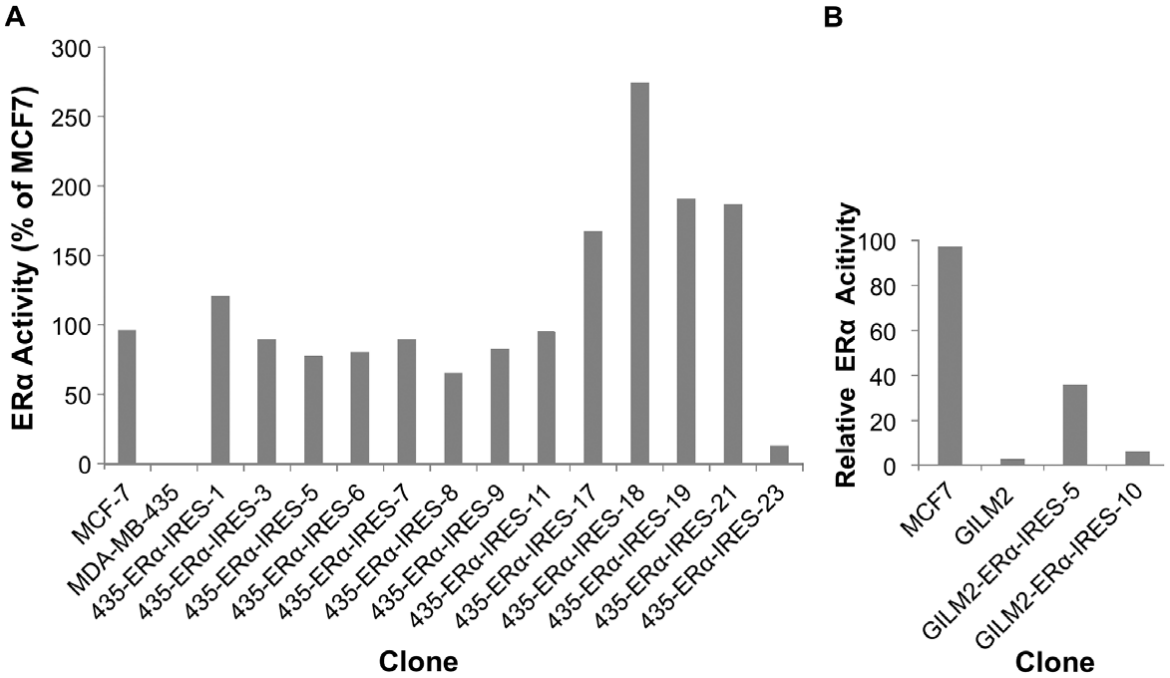
pERα-IRES MDA-MB-435 (A) & GILM2 transfectants (B): Dual-Luciferase reporter assay screening for ERα activity. Technical details as in the legend to Fig. 6.

Initially, we tested the clones for ERα expression by Western blot analysis. Fig. 11A & 11B show the results obtained with MDA-MB-435 clones. Importantly, similar to MDA-MB-231, most of the hygromycin B resistant clones of MDA-MB-435 (13 out of 13) and GILM2 (2 out of 4; data not shown) displayed some level of ERα expression. We next tested these clones for their ERα activity, by the dual luciferase reporter assay (Fig. 12). ERα activity was measured in 12 out of 13 MDA-MB-435 (Fig. 12A) and in two out of four of the GILM2 ERα-IRES clones (Fig. 12B).

**Figure 13:**
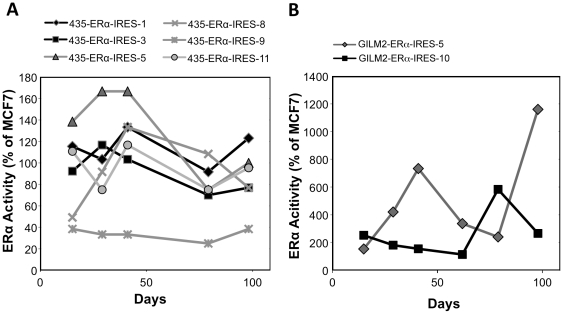
pERα-IRES MDA-MB-435 (A) & GILM2 transfectants (B): ERα activity over time. Technical details as in the legend to Fig. 6. ERα values were normalized to the values obtained at each time point with MCF-7 cells, taken as the 100%.

In order to evaluate the stability of ectopic ERα expression over time of each cell clone, dual-luciferase assays were performed over a relatively long time period for both cell lines (Fig. 13). Cell clones were kept under hygromycin B selection and in phenol red-free DMEM supplemented with 5% CSS for the entire period. They were exposed to estrogen only at the time of assay.

As it turned out, in the case of MDA-MB-435 derived ERα-IRES stable transfectants, 11 clones out of 12 retained at least 50% of their initial ERα activity (data not shown). As compared to the activity of MCF-7 cells which were assayed alongside the clones, at each time point, five of the six cell clones had at least 75% of MCF-7 ERα activity (Fig. 13A). In the case of the two GILM2 derived ERα-IRES expressing cell clones, there was at least retention of their initial ERα activity (Fig. 13B).

## Discussion

This project was aimed at establishing an efficient method for ERα complementation in various ERα-deficient cell lines. The generation of these complemented clones served as a counterpart control ingredient for synthetic lethality screening systems in ERα-deficient TN breast carcinoma cell lines.

Studies performed by others [17], [18], together with unpublished experiments performed in our laboratory in breast cancer BT549 cells and MDA-MB-231 cells (mentioned in Section B.1) have shown that the yield of cell transfectants expressing appreciable levels of ectopically mono-cistronic transduced ERα is very low (5–12.5%). Our attempts to generate such MDA-MB-231 stable transfectants under minimal exposure to the ERα ligand did not improve this low efficiency (Figs. 2–6). Moreover, stable ERα expression in the MDA-MB-231 cell line transfectants lasted for 130 days in only three out of the initial five “stable” clones monitored for prolonged periods (Fig. 6). We therefore decided to try setting up a modified system in which the fraction of ERα expressing cell clones would be higher and the expression perhaps more stable.

Based on the discovery of the EMCV IRES element by E. Wimmer's laboratory, researchers have started using IRES-containing bicistronic mammalian vectors to co-express multiple genes [11], [12], [26], [27]. Following that line, we chose the pIREShyg3 mammalian bicistronic expression vector. This vector is equipped with multiple cloning sites downstream of the strong cap-dependent CMV immediate early promoter and upstream of an intron fused to the IRES element which directs the translation of the dominant selectable marker-Hygromycin B resistance. Despite weaker translation from the downstream IRES element, the Hygromycin B resistance gene can be easily selected for. We cloned the ERα ORF into this bicistronic vector and then transfected it into the MDA-MB-231 breast carcinoma cell line. Maintaining the cells under phenol red-free DMEM supplemented with 5% CSS while selecting for Hygromycin B resistance, led to the isolation of nine clones. The ERα producing clones were identified by Western immunoblot analysis. All Hygromycin B resistant cell clones expressed the correct size ERα protein (Fig. 7). When assayed, the ERα protein turned out to be functionally active (Fig. 8). Importantly, nine out of the nine clones had high levels of ERα expression. Evidently, the selection for expression of the downstream Hygro^R^ gene had a protecting effect on the upstream ERα gene expression from the same (bicistronic) transcription unit.

The mRNAs of the ERα producing clones were tested in an RT-PCR assay, verifying the integrity of the bicistronic mRNA (Fig. 10). Yet, with regard to MDA-MB-231 parental cells, although we were able to obtain cell clones such as ERα-IRES-3, which retained significant activity over a period of at least 155 days, most of MDA-MB-231 IRES-ERα descendents had intermediate activity over time (Fig. 9). Nevertheless, this intermediate level of expression, alongside plentiful clones was sufficient to complete any screen or study required.

Following MDA-MB-231 cells, we attempted usage of the pIRES-ERα vector in other breast cancer cell lines: MDA-MB-435, and GILM2. We received high initial yields of ERα expression; 12 out of 13 clones for MDA-MB-435, and 2 out of 4 for GILM2 (Figs. 11 & 12). Most of these cell clones retained high activity for at least 98 days (Figs. 13a & 13b).

Noteworthy, usage of the Hygro^R^ selectable marker gene driven by the relatively strong promoter HSV TK, such as in the mono-cistronic ERα expressing vector pCMV-Bam-ERα-Hygro, led to only 3–4 stable MDA-MB-231 cell clones out of forty eight which express the ERα receptor (Lilach Wallerstein-Shomrony M.Sc. Thesis Tel Aviv University 2006). So the difference in the yield of stable ERα expressing cell clones between the bicistronic vector and the mono-cistronic pCDNA3-ERα cannot be due to the usage of a different selectable marker (Hygromycin^R^ vs. neo^R^) or a weaker promoter driving the selectable gene (CMV vs. SV40 early), respectively.

The proven ability of the bicistronic vector to generate multiple ERα expressing clones at very high yields, which for the most part retain stable expression upon further propagation, is the major point of this manuscript. We would like to suggest those who are encountering hardships in other ectopic gene expression systems, to adopt the usage of such bi- or multi-cistronic vectors.

Using the cell systems generated in MDA-MB-231, MDA-MB-435 and GIML2, we are now focusing our attempts on genetic synthetic lethality screenings [7]. These screenings entail a group of 100 human antiapoptotic/survival genes (known to be expressed in human breast cancers), and thereby promoting tumor growth and survival, as well as a lentiviral pool of shRNAs expressing clones targeted against all known human coding RNAs [7].
